# Galectin-9 and PSMB8 overexpression predict unfavorable prognosis in patients with AML

**DOI:** 10.7150/jca.53686

**Published:** 2021-05-19

**Authors:** Yongping Zhang, Song Xue, Qi Hao, Fuhong Liu, Wenqiu Huang, Jingbo Wang

**Affiliations:** Department of Hematology, Aerospace Center Hospital, Peking University Aerospace School of Clinical Medicine, Beijing 100049, China.

**Keywords:** acute myeloid leukemia (AML), Galectin-9 (Gal-9), bioinformatics analysis, proteasome subunit beta type-8 (PSMB8), biomarker, prognosis

## Abstract

Acute myeloid leukemia (AML) is a deadly heterogeneous hematologic malignancy. Despite the well-characterized genetic characteristics and new promising targeted therapies for AML, the clinical outcome remains suboptimal. Galectin-9 (Gal-9) is a good potential target due to its immunosuppressive capacity in inflammatory processes. In our study, we firstly performed a wide range of integrated bioinformatical approach to assess the importance of Gal-9 by analyzing the expression, potential function and prognostic impact in AML. The results indicated that Gal-9 is overexpressed in AML cells, especially when relapse after hematopoietic stem cell transplantation (HSCT) and predicts poor prognosis. Co-expression analysis showed Gal-9 has a strong positive correlation with proteasome subunit beta type-8 (PSMB8), which was also highly expressed in AML with poor prognosis, implying a synergy in cell survival, cell signaling and the development of AML. In summary, we have confirmed the overexpression of Gal-9 and its partner PSMB8 in AML and validated their importance as prognostic factors. We propose that Gal-9 and PSMB8 could be a promising molecular target for treatment of AML and may provide more combined treatment options, especially in patients with relapse after HSCT.

## Introduction

AML is a clinically and genetically heterogeneous disease that originates from hematopoietic stem and progenitor cells (HSPCs) characterized by differentiation blockade and clonal proliferation of immature blast cells reside in bone marrow, peripheral blood and extramedullary sites suppressing normal hematopoiesis^1^. It is the most common acute leukemia in adults with an incidence that increases with age 2. The standard of induction chemotherapy regimen consisting of cytarabine and anthracycline for AML was established over the last decades and remains profoundly unchanged today 1. Even though up to 85% younger patients (≤60 years) patients respond to induction chemotherapy and achieve complete remission, the most patients will eventually relapse within 3 years 1. Only about 25% AML patients survives 5 years or longer in spite of multidrug combined chemotherapy, molecular targeted therapies and HSCT 3. Prognosis is even worse in elderly patients (>60 years) and those who cannot tolerate standard induction chemotherapy, with a median survival of only 5-10 months and 5-year overall survival (OS) of 5% 1, 4, 5. Thus, there is an urgent need for the development of novel therapeutic approaches for AML.

One promising molecular target is the Gal-9, a member of the ninth member of the β-galactoside-binding soluble lectin family consists of two carbohydrate recognition domains linked by a peptide, and it was first identified as an eosinophil chemoattractant and activation factor 6, 7. The Gal-9 gene LGALS9 is localized in the short arm of human chromosome 17, which has several isoforms 8. Gal-9 is found in both the cytoplasm and extracellular regions 9 and expressed on immune cells, parenchymal cells and a variety types of tumor cells 10. Gal-9 has been shown to regulate many different biological functions such as inflammation, cell adhesion, cell proliferation, and cell apoptosis 8, 10, 11. Numerous studies have shown that the interactions between Gal-9 and its ligand, T cell immunoglobulin mucin-3 (Tim-3), negatively regulates T cell responses through promoting Th1, Th17 and cytotoxic T cells exhaustion, driving the expansion of myeloid-derived suppressor cells and FoxP3+T regulatory cells (Tregs) 11, 12. As Gal-9 was highly expressed on AML cells 10, which may result in tumorigenicity, tumor growth and escape from immunity and Gal-9 may be a biomarker of poor prognosis for AML. In this work, we firstly performed a wide range of integrated bioinformatical approach to assess the importance of Gal-9 by analyzing the expression, potential function and prognostic impact of Gal-9 in human AML.

## Methods

### Bioinformatics Mining for Identifying Gal-9 Expression

GEPIA (*http://gepia2.cancer-pku.cn*) and Oncomine (*https://www.oncomine.org/resource/main.html*) cancer databases were mined to determine the Gal-9 differential expression level between an AML group and a normal group. GEPIA is a newly developed interactive web server for analyzing the RNA sequencing expression data of 9736 tumors and 8587 normal samples from the TCGA and the GTEx projects, using a standard processing pipeline 13. The Oncomine database is a publicly accessible, online cancer microarray database that helps facilitate research from genome-wide expression analysis. The fold change was defined as >2 and the gene ranks in the top 10%, p value was set up at < 1E-4. The mRNA levels of Gal-9 in a series of cancers were analyzed by *CCLE* database *(https://portals.broadinstitute.org/ccle/about)*, which is an online encyclopedia of a compilation of gene expression, chromosomal copy number and massively parallel sequencing data from 947 human cancer cell lines, to facilitate the identification of genetic, lineage, and predictors of drug sensitivity.

### Membrane Immunostaining and Flow Cytometry

Mononuclear bone marrow cells were obtained from AML patients and healthy donors with informed consent. The eligible patients AML patients (excludes acute promyelocytic leukemia) were all hematological relapsed (Bone marrow blasts ≥5%; or reappearance of blasts in the blood) after chemotherapy or HSCT. 22 patients relapsed after HSCT, including 10 males and 12 females, with a median age of 29 (16-54) years old. 35 patients relapsed after chemotherapy, including 22 males and 13 females, with a median age of 41 (14-64) years old. Patients with complete remission and molecular relapse were excluded (Table 1).

Single-cell suspensions of mononuclear bone marrow cells were stained with the anti-galectin-9 antibody (BD Biosciences) and analyzed by flow cytometry using the Cell Quest software (FACSCalibur; BD Biosciences).

### Co-expressed Genes of Gal-9

The UALCAN database (*http://ualcan.path.uab.edu/*) was used to select the top 200 positively co-expressed genes of Gal-9, meanwhile the GEPIA dataset was applied to obtain top 200 co-expressed genes of Gal-9 for AML. The co-expressed genes obtained from the two databases were cross-referenced to obtain a cohort of 138 common co-expressed genes by a web tool *(http://bioinformatics.psb.ugent.be/webtools/Venn/)*.

### Functional and KEGG Pathway Enrichment Analysis

WebGestalt (WEB-based Gene SeT AnaLysis Toolkit) (*http://www.webgestalt.org/*) is a functional enrichment analysis web tool that reveals the biological meaning behind by entering a list of genes. Based on the candidate genes, gene ontology (GO) annotations can be divided into three categories: biological processes (BP), cellular components (CC) and molecular functions (MF) 14. The KEGG pathway database is used to identify biological pathways for co-expressed genes enrichment 15. We chose the top 10 terms to be the key pathways. False discovery rate (*FDR) <* 0.05 was set as the cut-off criterion.

### Protein-Protein Interaction Network Construction and Hub Genes Identification

Protein-Protein Interaction analysis (PPI) for co-expressed genes were performed by STRING database (*https://string-db.org/*) with an interaction score of >0.4 16, 17. Then, the Cytoscape software (version 3.8.0) was employed for visualizing molecular interaction networks. Each node is a gene, protein, or molecule, and the connections between nodes represent the interaction of these biological molecules, which can be used to identify interactions and pathway relationships between the proteins encoded by co-expressed gens in AML. The plug-in cytoHubba of Cytoscape was used to rank the genes by MCC method and the top 10 genes were determined as hub genes for further analysis.

### Gene Correlation and Survival Analysis in GEPIA

The real hub genes and Gal-9 were performed gene expression correlation analysis by using given sets of TCGA expression data in GEPIA. The correlation coefficient was determined by the Pearson method. Gal-9 was presented on the x-axis, and hub genes were represented on the y-axis for tumor vs normal tissue analysis. Moreover, we used the GEPIA database to perform survival analysis for Gal-9 and the hub genes.

### Statistical Analysis

Data in GEPIA databases, Oncomine databases and flow cytometry were analyzed by the student's t-test to compare the differential expression levels of Gal-9 mRNA or protein between two groups. Log-rank test was used for computing *P*-value in Kaplan-Meier univariate survival analyses. Pearson's correlation test was employed to evaluate the correlation between GAL-9 expression and hub gens. Fisher's exact test were performed to measure the gene enrichment in annotation terms and KEGG pathway. *P* < 0.05 was considered as statistically significant difference.

## Results

### Up-regulation of Gal-9 Expression in AML and High Gal-9 Expression

#### Predicts Poor Prognosis

**Figure 1:**
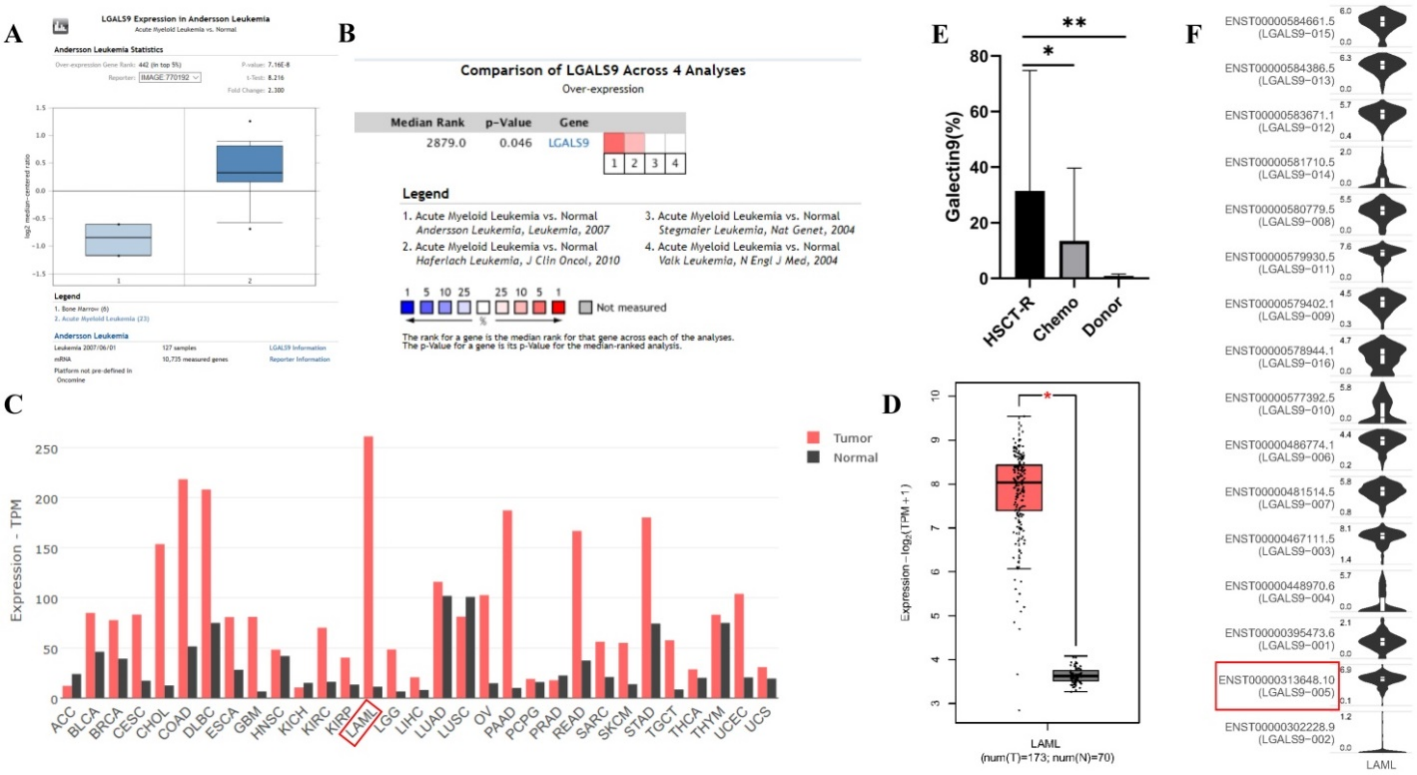
LGALS9 (Galectin-9) mRNA and protein expression was elevated in AML. (A) Box plots derived from gene expression data in *ONCOMINE* comparing expression of LGALS9 in normal and AML samples. The *p* value was set up at 0.01 and fold change was defined as 2. (B) Meta-analysis of LGALS9 mRNA expression levels across 4 analyses in Oncomine database. (C,D) The GEPIA database verified that LGALS9 gene expression was significantly upregulated in AML samples (LAML) (n=173) compared with normal samples (n=70), *P < 0.05. (E) Galectin-9 protein was highly expressed on the cell surface of AML cells, especially when relapse after HSCT compared with healthy donor derived mononuclear bone marrow cells and AML cells without HSCT in clinical practice. (F) LGALS9 has several isoforms, the mRNA expression level of LGALS9-005 ranked the highest among different protein coding types.

**Figure 2:**
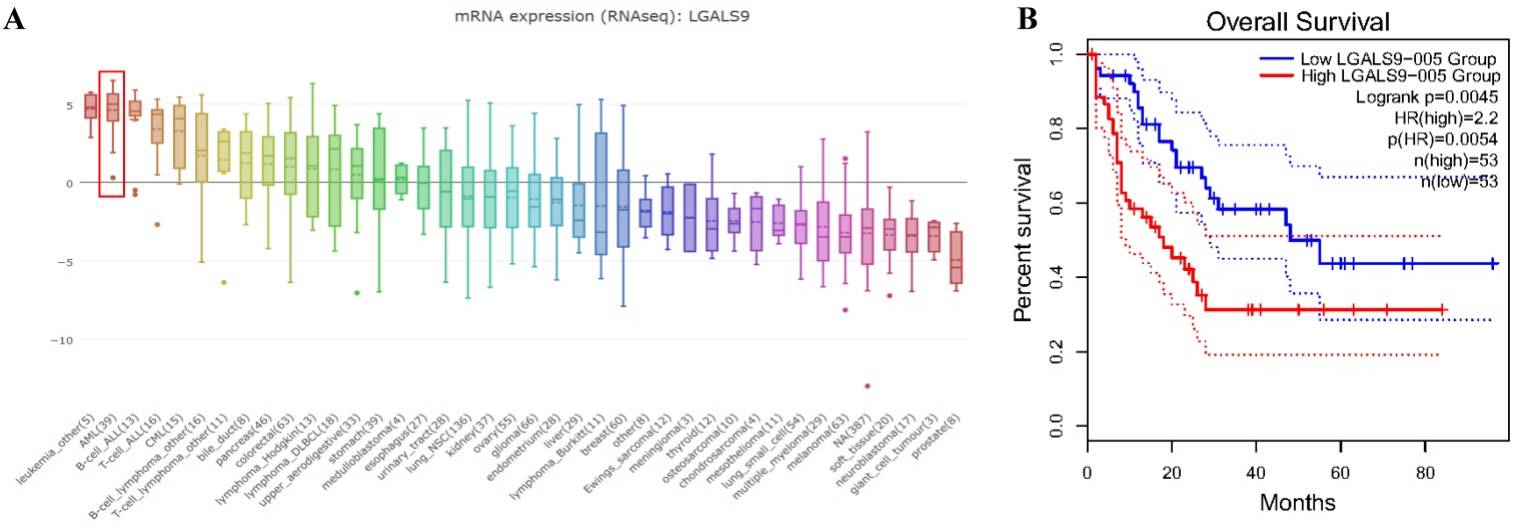
LGALS9 was distinctively high expressed AML cell lines from CCLE analysis and prognostic significances of LGALS9 gene expression. (A) The mRNA expression level of LGALS9 ranked the second highest in a variety of cancer cell line (shown in red frame). (B) LGALS9 overexpression predicts unfavorable overall survival in patients with AML.

We analyzed the expression profile of Gal-9 using both Oncomine and GEPIA database. The expression of Gal-9 was significantly higher in the AML group than that in the normal group (all *P*<0.05, Figure 1A-D). Gal-9 has several isoforms, the mRNA expression level of LGALS9-005 ranked the highest among different protein coding types (Figure 1F). For validation, we performed meta-analysis of Gal-9 expression in 4 analyses with threshold by p-Value ≤ 0.05, fold change ≥ 2 and top 10% gene rank in Oncomine database. As shown in Figure 1B, compared with that in normal bone marrows, Gal-9 was significantly upregulated in AML (*P* < 0.05, Figure 1B). In addition, *CCLE* analysis was consistent with that of *ONCOMINE* demonstrating that Gal-9 were distinctively up-regulated in AML cell lines (Figure 2A). In addition, Gal-9 protein was highly expressed on the cell surface of AML cells, especially when relapse after HSCT compared with healthy donor derived mononuclear bone marrow cells and AML cells without HSCT in clinical practice (Figure 1E; Table 1). These results implied that Gal-9 might play a unique role in the development of AML. Moreover, we used the GEPIA database to perform survival analysis for Gal-9 and found that increased expression of Gal-9 mRNA was significantly associated with poor overall survival (OS) in AML (Figure 2B).

### Functional and KEGG Pathway Enrichment Analysis Revealing Functional Association of Gal-9 with Immunoproteasome

**Figure 3:**
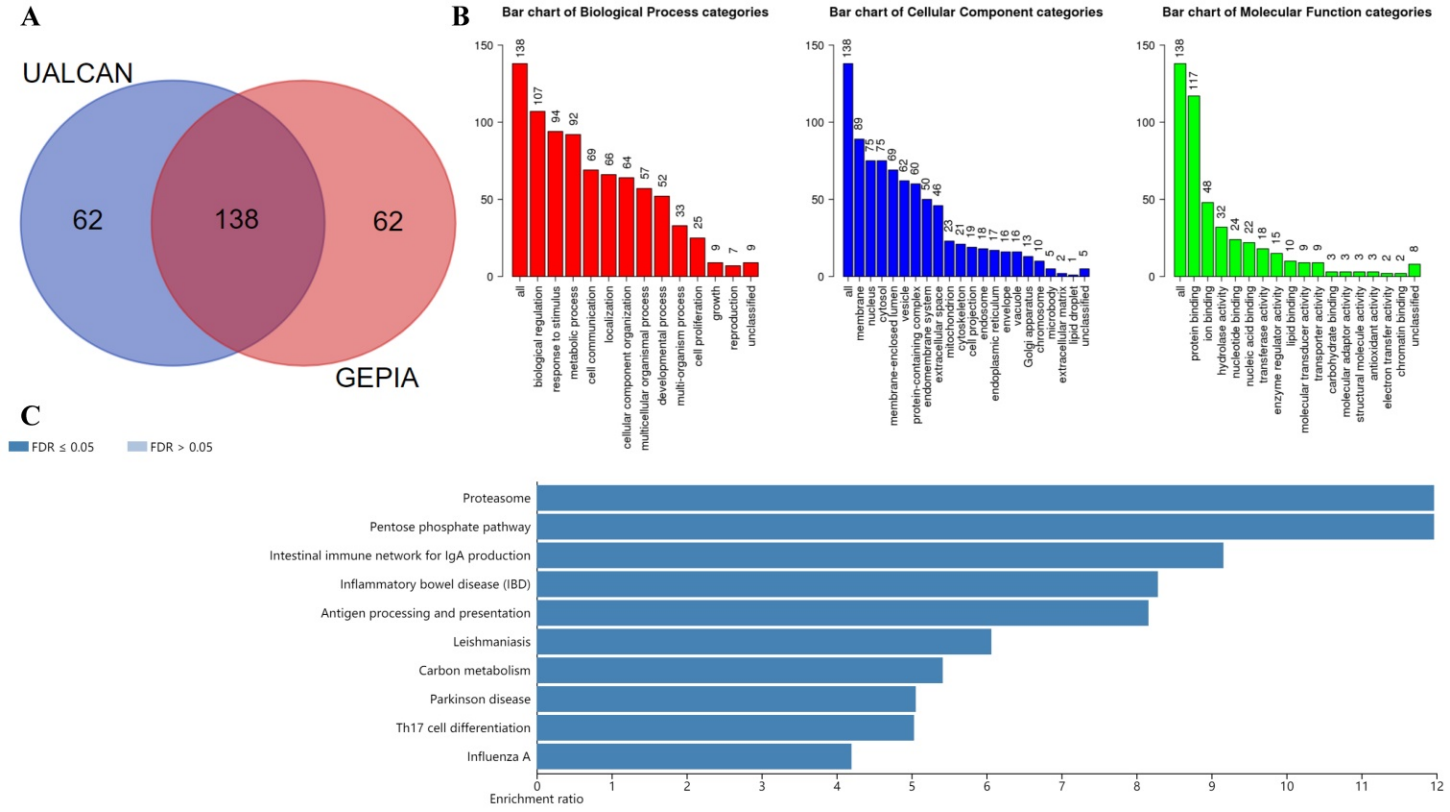
KEGG and GO enrichment analysis revealing functional association of LGALS9 with immunoproteasome. (A) Venn diagram represents the intersection of the top 200 positively corrected genes between the UALCAN database and the GEPIA database, 138 common co-expressed genes were obtained. (B) GO enrichment of co-expressed genes in biological process, cellular component and molecular function. (C) KEGG enrichment analysis of co-expressed genes with LGALS9. False discovery rate (*FDR) <* 0.05 was set as the cut-off criterion.

The UALCAN database was used to select the top 200 co-expressed genes of Gal-9. Meanwhile the GEPIA dataset was applied to obtain top 200 co-expressed genes of Gal-9 for AML. The co-expressed genes obtained from the two databases were cross referenced to obtain a cohort of 138 common co-expressed genes (Figure 3A). To analyze the biological classification of co-expressed genes, we used WebGestalt web tool for functional and pathway enrichment analysis. GO analysis indicated that the biological processes including biological regulation, response to stimulus, metabolic process, cell communication, localization, cellular component organization, multicellular organismal process, developmental process, multi-organism process, cell proliferation were significantly affected, consistent with enrichment in respective cellular locations and proposed molecular functions (Figure 3B). KEGG pathway enrichment of Gal-9 interactive genes showed that proteasome, antigen processing and presentation, inflammatory bowel disease, Parkinson disease, intestinal immune network for IgA production, carbon metabolism, pentose phosphate pathway, influenza A, Th17 cell differentiation, Leishmaniasis were the most enriched pathways (Figure 3C). Collectively, these data suggest an essential role of Gal-9 in regulating many different biological functions such as inflammation, cell adhesion, cell proliferation, and cell apoptosis in AML. Interestingly, the function of Gal-9 in AML may be closely related to immunoproteasome.

### Gal-9 PPI Network Construction and Analysis of 10 Hub Genes

**Figure 4:**
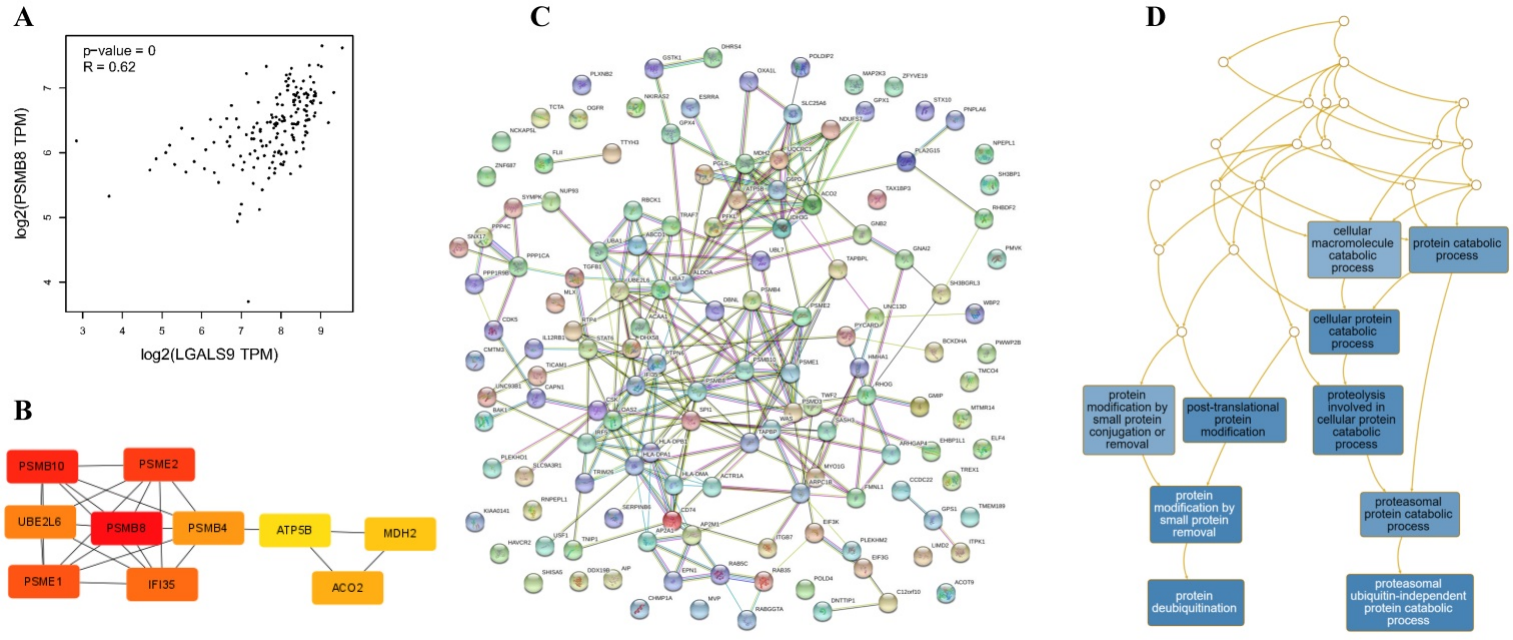
Construction of PPI network of LGALS9 positive-correlation genes and analysis of hub genes. (A) Correlation between LGALS9 and PSMB8 mRNA expression determined using GEPIA database. (B) The top ten hub genes were identified using cytoHubba tool kits in Cytoscape. (C) Clustering analysis of LGALS9 co-expressed genes by STRING tools. (D) The KEGG pathway analysis of hub genes using WebGestalt web tool.

**Figure 5:**
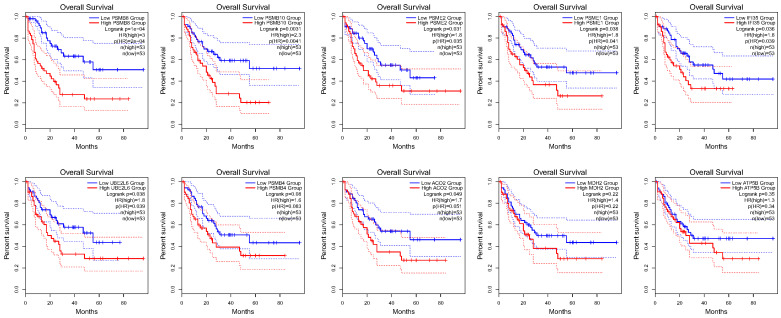
Over survival analyses of hub genes in AML using GEPIA database. 7 of the10 hub genes exhibited poorer overall survival rate in higher expression groups.

Using the STRING database, the co-expressed 138 genes were constructed into a protein-protein network (Figure 4C). The top ten genes showing significant interaction including the PSMB8, PSMB10, PSME2, PSME1, IFI35, UBE2L6, PSMB4, ACO2, MDH2, ATP5B were identified as potential hub genes according to the MCC score generated by CytoHubba plug-in (Figure 4B). The KEGG pathway analysis of hub genes were further performed using WebGestalt web tool. Particularly, proteasomal ubiquitin-independent protein catabolic process and protein deubiquitination were the most enriched pathways, suggesting that they may participate in the protein anabolism required for cell survival and cell signaling (Figure 4D). Furthermore, the overall survival of hub genes was analyzed using Kaplan-Meier curve through the GEPIA database. 7 of these 10 hub genes exhibited poorer overall survival rate in higher expression groups (Figure 5). Amongst these hub genes, PSMB8 may be the most attractive target in AML. PSMB8 was highly expressed in AML and with poor prognosis (Figure 5). What's more, GEPIA correlation analysis showed that Gal-9 and PSMB8 had high correlation coefficients (Pearson's correlation = 0.62) (Figure 4A). These data suggest that Gal-9 and immunoproteasome may play a cooperative role in the development of AML.

## Discussion

AML is one of the most common lethal hematologic malignancies 1. Despite well-characterized molecular and genetic characteristics, targeted therapies for AML have yet to lead to significantly improved clinical outcomes 1. The pathogenesis, diagnosis and treatment of AML remains the matter of intense research currently.

This study was the first to show the mRNA expression and prognosis of Gal-9 in AML, although other studies have reported the role of Gal-9 in tumorigenesis, development, metastasis and potential therapy of several cancers 7, 8. As a member of the galectin family, more and more research revealed that Gal-9 is a negative regulator of tumor immune responses by promoting Th1, Th17 and cytotoxic T cells exhaustion, driving the expansion of myeloid-derived suppressor cells and FoxP3+Tregs when interactions with its ligand Tim-3 8, 10, 11. Gal-9 plays an essential role in regulating inflammation, cell adhesion, cell proliferation, and cell apoptosis 8, 10, 11. Our study has revealed that Gal-9 was overexpressed in AML, what's more, Gal-9 protein was highly expressed on the cell surface of AML cells, especially when relapse after transplant compared with healthy donor derived mononuclear bone marrow cells and AML cells without HSCT in clinical practice. The Gal-9 associated co-expressed genes are primarily involved in immune response and antigen processing and presentation, these data suggest that Gal-9 may play an important role in immune escape of AML and consistent with the indication of poor prognosis in AML patients. Thus, targeting Gal-9 may be a promising strategy for therapeutic intervention against AML. So far, some strategies appear to be promising in abrogating galectin-mediated effects. These include: (1) neutralization of galectin-binding through blocking antibodies against galectins 18, (2) competitive inhibition of galectin binding via synthetic analogs of galectin carbohydrate-binding determinants 19, (3) modification of the glycosylation profiles necessary for galectin binding on anti-tumor immunocytes through fluorinated analogs of glucosamine 7, 20. Recently, a Phase IB (NCT03066648) clinical trial was undertaken to investigate the therapeutic effects on blocking galectin-9/TIM-3 pathway with anti-TIM-3 antibody, about one-third newly diagnosed and relapse/refractory AML patients achieved complete or partial response 21. Indeed, treatment with anti-Tim-3 alone has little or no effect because of the complex and multiple biological features of AML. Combined treatment is still the future direction for AML.

PSMB8 is a member of the proteasome β-type family, which is also named the large multifunctional protease 7 (LMP7), encodes β5i which often functions as a subunit of the immunoproteasome 22. PSMB8 encodes β5i which performs as one of the catalytic subunits of immunoproteasome and takes part in immunological reaction via 26S proteasome mediated degradation 22. PSMB8 plays an important role in promoting cell survival and progression of the leukemia through PSMB8-mediated PI3K/AKT pathway activation 23 and constitutive nuclear factor κB (NF-κB) signaling, while NF-κB is constitutively active in leukemic stem cells (LSCs), but not in normal hematopoietic progenitor cells 24-26. The expression of PSMB8 was remarkably correlated with Ki-67, which is always considered as a cancer marker 27. These findings indicate that PSMB8 may be used as a novel prognostic factor and therapeutic target of AML.

In this study, we found PSMB8 was highly expressed in AML with poor prognosis. The positive correlation of Gal-9 and PSMB8 in AML may indicate a synergy in cell survival, cell signaling and the development of AML, co-targeting these two genes might be an efficient way to treat AML.

For most patients with AML, allogeneic hematopoietic cell transplantation is the only curative treatment, however relapse is the common cause of death and its incidence and outcome have not significantly improved over the last decades 28. The relapse of AML after HSCT is associated with immune escape. The mechanisms of immune evasion are complex and diverse, mainly including abrogation of leukemia cell recognition due to loss of HLA genes, immunosuppression by immune-checkpoint ligand expression, production of anti-inflammatory factors, release of metabolically active enzymes, loss of proinflammatory cytokine production, and acquisition of novel driver mutations that promote leukemia outgrowth 29, 30. In our study, Gal-9 protein was highly expressed on the cell surface of AML cells, especially when relapse after transplant, indicating its important role in immune escape.

In summary, we have confirmed the overexpression of Gal-9 and its partner PSMB8 in AML and validated their importance as prognostic factors. We propose that Gal-9 and PSMB8 could be a promising molecular target for treatment of AML and may provide more combined treatment options, especially in patients with relapse after HSCT.

## Figures and Tables

**Table 1:** Clinic parameters of AML patients and healthy donors

Parameter	HSCT-R (n=22)	Chemo-R (n=35)	Donors (n=15)
Age (years)	29 (16-54)	41 (14-64)	36 (6-64)
Sex	10M; 12F	22M; 13F	8M; 7F
Blast counts (%) (±SD), P=0.20	53.8 (±30.4)	41.5 (±28.8)	-
Mean Gal-9 counts (%) (±SD)	31.4(±43.3)	13.5 (±26.1)	0.9 (±0.64)

AML, acute myeloid leukemia; HSCT, hematopoietic stem cell transplantation; R, relapsed; Chemo, chemotherapy; M, male; F, female; Gal-9, Galectin-9; SD, standard deviation.
